# Does timing matter in radiotherapy of hepatocellular carcinoma? An experimental study in mice

**DOI:** 10.1002/cam4.4277

**Published:** 2021-09-20

**Authors:** Soha A. Hassan, Amira A. H. Ali, Dennis Sohn, Ulrich Flögel, Reiner U. Jänicke, Horst‐Werner Korf, Charlotte von Gall

**Affiliations:** ^1^ Institute of Anatomy II, Medical Faculty Heinrich‐Heine‐University Düsseldorf Germany; ^2^ Zoology Department Faculty of Science Suez University Suez Egypt; ^3^ Department of Anatomy and Embryology Faculty of Medicine Mansoura University Mansoura Egypt; ^4^ Laboratory of Molecular Radiooncology, Clinic and Policlinic for Radiation Therapy and Radiooncology Medical Faculty of Heinrich‐Heine‐University Düsseldorf Germany; ^5^ Department of Molecular Cardiology Heinrich‐Heine‐University Düsseldorf Germany; ^6^ Institute of Anatomy I Medical Faculty Heinrich‐Heine‐University Düsseldorf Germany

**Keywords:** clock genes, hepatocellular carcinoma, Ki67, radiotherapy, γ‐H2AX

## Abstract

This study investigates whether a chronotherapeutic treatment of hepatocellular carcinoma (HCC) may improve treatment efficacy and mitigate side effects on non‐tumoral liver (NTL). HCC was induced in *Per2*::*luc* mice which were irradiated at four time points of the day. Proliferation and DNA‐double strand breaks were analyzed in irradiated and nonirradiated animals by detection of Ki67 and γ‐H2AX. Prior to whole animal experiments, organotypic slice cultures were investigated to determine the dosage to be used in whole animal experiments. Irradiation was most effective at the proliferation peaks in HCC at ZT02 (early inactivity phase) and ZT20 (late activity phase). Irradiation effects on NTL were minimal at ZT20. As compared with NTL, nonirradiated HCC revealed disruption in daily variation and downregulation of all investigated clock genes except *Per1*. Irradiation affected rhythmic clock gene expression in NTL and HCC at all ZTs except at ZT20 (late activity phase). Irradiation at ZT20 had no effect on total leukocyte numbers. Our results indicate ZT20 as the optimal time point for irradiation of HCC in mice at which the ratio between efficacy of tumor treatment and toxic side effects was maximal. Translational studies are now needed to evaluate whether the late activity phase is the optimal time point for irradiation of HCC in man.

## Graphical abstract

To elucidate whether a chronotherapeutic approach may improve the efficacy of radiotherapy for hepatocellular carcinoma (HCC) which was experimentally induced in mice by defining the optimal time point at which the HCC is more radiosensitive, while the surrounding non‐tumoral liver is more radioresistant to the damaging effects. Our results from HCC‐bearing mice irradiated at four different time points allowed us to define ZT20 (late activity phase) as an optimal time point to apply radiotherapy because of least toxicity and similar efficacy against HCC as compared to other tested ZTs. Translational studies are required to confirm the findings for HCC patients.

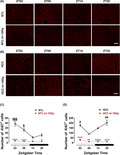

## 1 INTRODUCTION

Hepatocellular carcinoma (HCC) occupies the fourth cause of cancer death worldwide with mortality rate of 8.2% (782,000 deaths) and 841,080 new cases in 2018.^1^ HCC is characterized by high malignancy, fast progression, invasion, and metastasis. Moreover, HCC is highly resistant to antimitotic therapies.^2^ Due to this resistance, the antimitotic chemotherapies (e.g., sorafenib) are recently replaced by immunotherapy alone (e.g., immune checkpoint inhibitors; ICIs) or in combination with different agents (e.g., multi‐tyrosine kinase inhibitor) for the HCC treatment. However, these combinations have severe side effects which may impair life quality of patients and interrupt the treatment.^3^, ^4^

Localized radiotherapy including stereotactic radiation therapy and selective internal radioembolization as well as radiation in combination with immuno‐ or chemotherapy are widely used for the HCC treatment with varying stages.^5^, ^6^, ^7^, ^8^ However, many precautions should be taken into consideration when delivering the radiotherapy due to the increased risk of radiation‐induced liver damage which follows the hepatic radiotherapy.^9^, ^10^ Thus, an important question is whether the application of chronotherapy might improve the efficacy of radiotherapy for HCC.

In a recent study with a mouse model of HCC (double transgenic c‐myc/TGFα mice), time‐dependent differences were shown in proliferation rate as well as DNA damage/repair mechanisms between the HCC and the surrounding non‐tumoral liver (NTL).^11^ These results suggest that the efficacy and side effects of any antimitotic therapy for HCC may depend on proper timing and define the optimal time point for antimitotic therapies may help to improve the efficacy of HCC treatment. Such a chronotherapeutic approach has been taken in humans for other tumors such as brain metastases, rectal, and cervical cancers but not for HCC.^12^

To test the potential value of a chronotherapeutic approach, we investigated the effect of irradiation at four different *Zeitgeber* time (ZT) points in a diethylnitrosamine (DEN)‐induced HCC mouse model, which closely mimics human HCC, in view of the multistage development of the liver tumors over months prior for them to become established and to progress. HCC was induced in *Per2*::*luc* mice previously used to evaluate the effects of x‐rays on the molecular clock in normal liver.^13^ In order to evaluate the response to radiotherapy, Ki67 was used as a marker for proliferation and γ‐H2AX as a marker for DNA‐double strand breaks (DSBs) in HCC and NTL. Ki67 is expressed during the G2/M phase of the cell cycle, which is the most critical target phase for radiotherapy.^14^ In HCC, the expression of Ki67 is established as an indicator for the response to antimitotic drugs (e.g., tivantinib).^15^ In addition, it is well known that during proliferation cells become more sensitive to DNA damage induced by cancer therapies.^16^ Thus, γ‐H2AX, a histone which accumulates in the damaged sites of DNA‐DSBs to start the DNA repair process,^17^ is used as an indicator for the sensitivity of tumors and the surrounding healthy tissues to the treatment protocols and helps to control the dose and the efficacy of radiotherapy.^17^ In HCC, γ‐H2AX was recently used as a marker to predict the efficacy of a combined sorafenib treatment with radiation (RT‐SOR).^8^

To determine the dose to be used in whole animal experiments, organotypic slice cultures (OSC) were irradiated with two different doses (2 and 10 Gy) at four different circadian time points and Ki67 and γ‐H2AX immunoreactive cells were analyzed.

Proliferation rate, DNA damage/repair mechanism and the sensitivity to antimitotic treatments are controlled by the molecular clockwork^18^, ^19^ which is based on clock genes that interact through positive and negative transcription‐translation feedback loops.^20^, ^21^ The transcription factors CLOCK and BMAL1 represent the positive elements in the loops and activate the expression of *Per* (*Per1* and *Per2*) and *Cry* (*Cry1* and *Cry2*) genes which form PER/CRY complexes representing the negative elements.^21^ This molecular clockwork controls the expression of more than 3000 so‐called clock‐controlled genes and, thus, rhythmic cell and organ functions. Disruption or downregulation of clock gene expression leads to genomic instability which increases the cellular proliferation rate and thus promotes carcinogenesis.^2^, ^22^ In a previous study, expression of *Per2* and *Cry1* was significantly lower and showed an altered rhythm in HCC.^11^ To date, little is known about the time‐dependent effects of radiotherapy on the molecular clockwork. Therefore, we analyzed whether radiotherapy at different ZTs affects the molecular clockwork in HCC and NTL.

Hematopoiesis is one of the most sensitive systems in the body to radiotherapy and reduction of white and red blood cells is one of the most common side effects of the radiotherapy.^23^, ^24^ Thus, blood cells of HCC‐bearing mice with and without irradiation at different ZTs were analyzed as an additional readout for the side effects of the radiotherapy.

## 2 MATERIALS AND METHODS

### 2.1 Experimental animals and HCC induction

Male transgenic *Per2*::*luc* mice on a C57BL6/J background were used according to accepted standards of humane animal care and federal guidelines and Directive 2010/63/EU of the European Union. All experiments were approved by the Regierungspräsidium Darmstadt and the Landesamt für Natur, Umwelt und Verbraucherschutz NRW (Reference number: AZ 81‐02.04.2018‐A146). At the age of 2 weeks, the mice were injected intraperitoneally between ZT02 and ZT04 with a single dose of DEN (10 mg/kg body weight; Sigma Aldrich) to induce HCC. Phenobarbital (PB) (Luminal, Desitin) was chronically administered in drinking water with a concentration of 0.05% to accelerate the HCC induction. Food and water containing PB were supplied *ad libitum*. All mice were kept under the standard light–dark (LD) cycle (12:12). ZT00 defines the onset of the light phase. All experiments during the dark phase were performed under dim red light. At the age of 7–10 months, HCC presented either as a single large tumor or as multiple small tumors (Figure S1). Tumor development was screened via magnetic resonance imaging and post mortem inspection.

### 2.2 Magnetic resonance imaging

For magnetic resonance imaging, mice were anesthetized with 1.5% isoflurane in a water‐saturated gas mixture of 20% oxygen in nitrogen applied at a rate of 75 ml/min by manually restraining the animal and placing its head in an in‐house‐built nose cone. Respiration was monitored with a pneumatic pillow positioned at the animal's back. Vital function was acquired by using an M1025 system (SA Instruments) to synchronize data acquisition with respiratory motion. Animals were placed within the resonator so that in z‐direction (30 mm) the field‐of‐view (FOV) covered the abdomen from just below the diaphragm down to the pelvis.

Data were recorded on a Bruker AvanceIII 9.4 Tesla Wide Bore (89 mm) nuclear magnetic resonance spectrometer (Rheinstetten) operating at a frequency of 400.13 MHz for 1H. Experiments were carried out using a Bruker microimaging unit (Micro 2.5) equipped with actively shielded gradient sets (capable of 1.5 T/m maximum gradient strength and 150 μs rise time at 100% gradient switching), a linear 1H 25‐mm birdcage resonator, and Paravision 5.1 as operating software.

Liver tumors were determined by acquisition of images with a respiratory‐gated 2D 1H multi‐slice fast low angle snapshot (FLASH) gradient‐echo sequence exploiting the native tissue contrast between NTL and tumor tissue (see Figure S2). Data were taken from a FOV of 25.6 × 25.6 mm^2^ with a spatial resolution of 100 × 100 µm^2^ (TE, 1.62 ms; TR, 111.52 ms; slices, 16; slice thickness, 1 mm; averages, 1, acquisition time, 15 s).

### 2.3 Irradiation of organotypic slice cultures

Mice with HCC were sacrificed at ZT02. The liver was dissected under sterile conditions and stored quickly in ice‐cold storage solution (MACS tissue storage solution; Miltenyi Biotec). NTL and HCCs were sliced separately into 600 μm thick sections using a Krumdieck tissue chopper (TSE Systems; Bad Homburg) and kept in ice‐cold sterilized Dulbecoo's phosphate‐buffered saline (Gibco by Life Technologies). Then, the slices were transferred to cell culture inserts (0.4 μm pores; Falcon) which were inserted in six‐well plates filled with 1 ml pre‐warmed culture medium modified according to previously published protocol.^25^ The medium consisted of DMEM, supplemented with 10% fetal bovine serum, 100 U/ml penicillin, 0.1 mg/ml streptomycin, 10 mmol/l HEPES, 1 mg/ml insulin, 8 mg/ml ascorbic acid, and 20 mmol/l sodium pyruvate. The slices from the NTL and HCCs of each mouse were randomly divided into three groups, one nonirradiated and two for irradiation with different doses, and placed in 12 different plates. All slices were cultured in an incubator under constant conditions of 37℃ and 5% CO_2_. On the next day, at 05:00 AM, the medium was changed and this time point was defined as CT00. Two hours after the medium change (CT02), the plates were removed from the culturing conditions and transferred to the irradiation lab in a cooler to reduce the possible changes in the ambient temperature. The slices were irradiated at four different CTs (CT02, CT08, CT14, and CT20) with two different doses, 2 Gy (at 175 kV and 15 mA, for about 2 min) and 10 Gy (at 175 kV and 15 mA, for about 10 min), using Gulmay RS225 x‐ray system (X‐Strahl). Nonirradiated slices were transported to the irradiation lab but did not receive irradiation. Within 1 h after irradiation, the slices were returned to regular culturing conditions. The slices were harvested after 48 hours at the same CTs used for irradiation. For immunohistochemistry, the slices were fixed in 4% paraformaldehyde (PFA) in phosphate‐buffered saline (0.1 M PBS, pH 7.4) for 12 h and then cryoprotected with gradually increasing concentrations of sucrose in PBS (15% and 30%). Then the slices were cut into 10 μm thick serial sections using a cryostat (Leica CM).

### 2.4 Irradiation of mice and *ex vivo* analyses

Forty‐eight HCC‐bearing mice (7–10 months) were used for ex vivo investigations and randomly divided into two groups: the first group comprised 24 animals which were irradiated with a dose of 10 Gy (irradiated group) at four different ZTs (ZT02, ZT08, ZT14, and ZT20). The second group comprised 24 mice with HCC which were transported to the irradiation lab together with the animals of the irradiated group at the same ZTs but they were not subjected to irradiation (nonirradiated group) to omit the effect of transportation.

For irradiation, the mice were deeply anesthetized by intraperitoneal injection with a mixture of ketamine (100 mg/kg body weight; Inresa) and xylazine (10 mg/kg body weight, Rompun 2%; Bayer Leverkusen) and then the whole animal's body irradiated with a dose of 10 Gy by fixing the animals on a styrofoam plate so that their ventral side was exposed to the irradiation source. Exposure with 10 Gy irradiation was performed as described above.

After 48 hours, the irradiated and nonirradiated mice (*n* = 6/ZT in each group) were sacrificed at the same ZTs used for irradiation. Blood was collected from the right atrium in EDTA blood tubes and quickly mixed to avoid coagulation. In addition, the blood cells were analyzed in a control group which did not receive any treatment. The complete blood counts were measured automatically using Scil Vet abc, animal blood count machine (Viernheim).

Each group of mice was randomly divided into two subgroups (*n* = 3/ZT), which were used for either immunohistochemistry or qPCR analysis. For immunofluorescence, the animals were anesthetized as mentioned above and then perfused transcardially with NaCl (0.9%) for 1 min followed by approximately 100 ml 4% PFA in PBS for 15 min. NTL and HCCs were excised and separated by a scalpel. Then the tissues were postfixed for 2 h in 4% PFA in PBS, cryoprotected with gradually increasing concentrations of sucrose in PBS (10%, 20%, and 30%) and cut into 12 μm thick serial frozen sections using a cryostat.

For qPCR, the mice were sacrificed and NTL and HCCs were freshly dissected, rapidly snap frozen in liquid nitrogen and stored at −80℃ until further use.

To demonstrate the effects of irradiation on the number of Ki67 and γ‐H2AX immunoreactive (+) cells, relative expression of clock genes and complete blood counts, we compared the nonirradiated NTL and HCC with the irradiated NTL and HCC at each ZT separately. The experimental design is shown in Figure S3.

### 2.5 Immunofluorescence

Sections from OSC and ex vivo samples were incubated with normal goat serum (1:20) diluted in PBS with 0.3% Triton (PBST) for 1 h at room temperature (RT) to minimize non‐specific staining. Then the sections were incubated with the primary antibodies against Ki67 (1:200, #KI6891C01, DCS, Hamburg, Germany) or against γ‐H2AX (1:200, #2577; Cell Signaling Technology) overnight at RT. The primary antibodies were diluted in 1% bovine serum albumin in PBST. On the next day, sections were incubated with secondary goat anti rabbit antibodies (Alexa Fluor 568 for Ki67 or Alexa Fluor 488 for γ‐H2AX) in PBS (1:250; Life Technologies) for 1 h in darkness at RT. For negative control, the primary antibodies were omitted and sections were only incubated with the secondary antibodies. For nuclear staining, all sections were incubated with Hoechst dye diluted in PBS (1:10,000) for 10 min in darkness at RT. The sections were then covered with fluorescent mounting media (Fluoromount‐G; Southern Biotech).

Sections were analyzed using a Keyence BZ‐X800 series microscope (Keyence) using x20 objective and the settings were kept constant for each staining. Six representative images at least from each animal/time point/group were analyzed and averaged. The number of immunoreactive (+) cells was counted by an investigator blind to the treatment. The number of positive cells was counted manually in each image in a total area = 0.4 mm^2^.

### 2.6 qPCR

Total RNA from ex vivo samples was extracted using a RNeasy Plus Universal Mini Kit (Qiagen). Total RNA concentration and purity were measured using a Nano‐Drop spectrophotometer. A RevertAid First Strand cDNA Synthesis Kit (Thermo Scientific) was used for the synthesis of the cDNA from 1 µg RNA. Primers for the clock genes *Per1*, *Per2*, *Cry1*, *Cry2*, *Clock*, *Bmal1*, and *Rev*‐*erbα* (Sigma Aldrich, Table 1) and the housekeeping gene, β‐actin, were validated using conventional PCR and gel electrophoresis. qPCR was performed using Step One Plus (Applied Biosystems) and SYBR GREEN (Kapa Abi‐Prism). The relative mRNA expression of the clock genes, normalized to the housekeeping gene, was calculated according to Pfaffl method.^26^

**TABLE 1:** List of primer sequences used in qPCR

Gene	Primer sequence
β‐Actin F	5′‐GGCTGTATTCCCCTCCATGC‐3′
β‐Actin R	5′‐CCAGTTGGTAACAATGCCATGT‐3′
mPer1 F	5′‐TGG CTC AAG TGG CAA TGA GTC‐3′
mPer1 R	5′‐GGC TCG AGC TGA CTG TTC ACT‐3′
mPer2 F	5′‐CCAAACTGCTTGTTCCAGGC‐3′
mPer2 R	5′‐ACCGGCCTGTAGGATCTTCT‐3′
mCry1 F	5′‐CTT CTG TCT GAT GAC CAT GAT GA‐3′
mCry1 R	5′‐CCC AGG CCT TTC TTT CCA A‐3′
mCry2 F	5′‐AGG GCT GCC AAG TGC ATC AT‐3′
mCry2 R	5′‐AGG AAG GGA CAG ATG CCA ATA G‐3′
mClock F	5′‐CAC CGA CAA AGA TCC CTA CTG AT‐3′
mClock R	5′‐TGA GAC ATC GCT GGC TGT GT‐3′
Bmal F	5′‐GTA GAT CAG AGG GCG ACA GC‐3′
Bmal R	5′‐CCT GTG ACA TTC TGC GAG GT‐3′
Rev‐erbα F	5′‐GGT GCG CTT TGC ATC GTT‐3′
Rev‐erbα R	5′‐GGT TGT GCG GCT CAG GAA‐3′

### 2.7 Statistical analysis

Statistics were calculated using Graph Pad Prism 8 software. Significant differences for rhythmic patterns in NTL and HCC were evaluated by RM one‐way analysis of variance (ANOVA) and ordinary one‐way analysis of variance (ANOVA) followed by *Tukey's* test for multiple comparisons between different time points. Two‐way ANOVA was used to validate differences according to time and treatment followed by *Sidak's* test for multiple comparisons between groups. The results were represented as mean ± standard error of the mean (SEM) and were regarded as significant at *p *< 0.05.

## 3 RESULTS

For the ex vivo experiments reported here, a dose of 10 Gy was selected based on the results from OSC which are presented in the supplementary material (Figures S4, S5).

### 3.1 Ki67 and γ‐H2AX in HCC and NTL in mice without and with irradiation (10 Gy) at four different ZTs (ex vivo)

**FIGURE 1:**
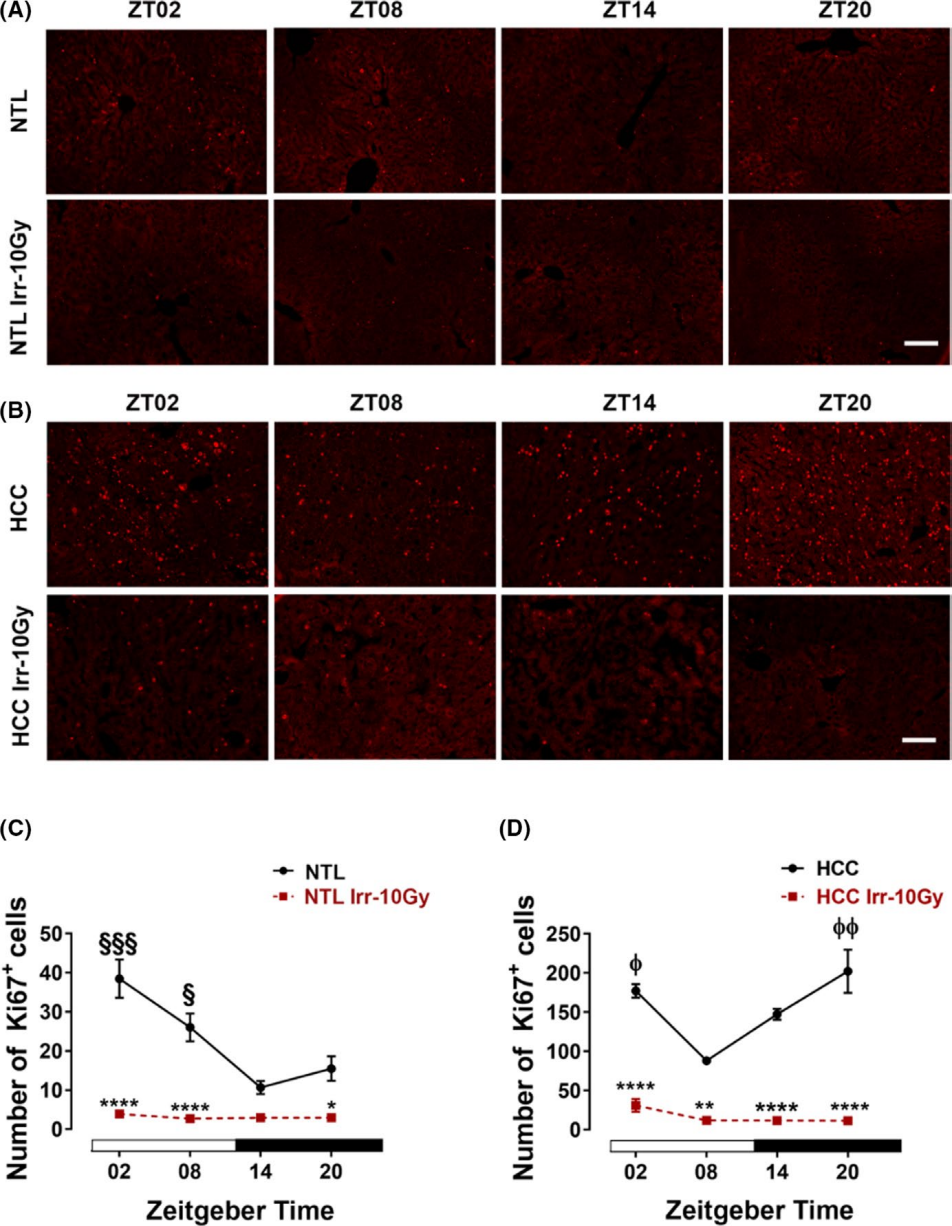
Ki67 in ex vivo samples of hepatocellular carcinoma (HCC) and non‐tumoral liver (NTL) with or without irradiation. At different *Zeitgeber* times (ZT00 = the onset of the light phase), mice were irradiated (Irr‐10 Gy) (*n* = 3/time point) or handled similarly but not irradiated. Forty‐eight hours later, mice were sacrificed at the same ZTs. Representative photomicrographs of Ki67 immunoreaction in NTL (A) and HCC (B). Quantification of Ki67 immunoreactive (+) cells in NTL (C) and HCC (D). Plotted are the mean numbers ± SEM of immunoreactive (+) cells. White and black bars indicate the light and dark phases, respectively. §: *p* < 0.05; §§§: *p *< 0.001 differences between this ZT and ZT14. φ: *p* < 0.05; φφ: *p* < 0.01 differences between this ZT and ZT08. *: *p* < 0.05; **: *p* < 0.01; ****: *p* < 0.0001 differences between the nonirradiated and irradiated group. Scale bars, 100 μm

In nonirradiated NTL, the number of Ki67+ cells showed one peak at ZT02 which was significantly different from the trough at ZT14 (*p *< 0.001, Figure 1A,C). In HCC, the number of Ki67+ cells was higher than in NTL at all ZTs (ZT02, 14, 20, *p *< 0.0001; ZT08, *p *< 0.01), but in contrast to the NTL, the number of Ki67+ cells showed two peaks, one during the early light phase (ZT02, *p *< 0.05) and the second in the late dark phase (ZT20, *p *< 0.01) as compared with the trough at ZT08 (Figure 1D).

Irradiation (10 Gy) resulted in a decrease in the number of Ki67+ cells. In NTL, the highest effect of irradiation was observed after irradiation during the light phase (ZT02, 89.8%; ZT08, 60.6%, *p *< 0.0001). During the dark phase, irradiation had little effects (ZT14, 20%; ZT20, 32.6%, *p *< 0.05) (Figure 1A,C). In HCC, irradiation resulted in a significant decrease in Ki67+ cells at all ZTs (ZT02, 72.3%; ZT08, 37.7%; ZT14, 67.1%; with the strongest decrease observed at ZT20, 94.3%) (Figure 1B, D).

**FIGURE 2:**
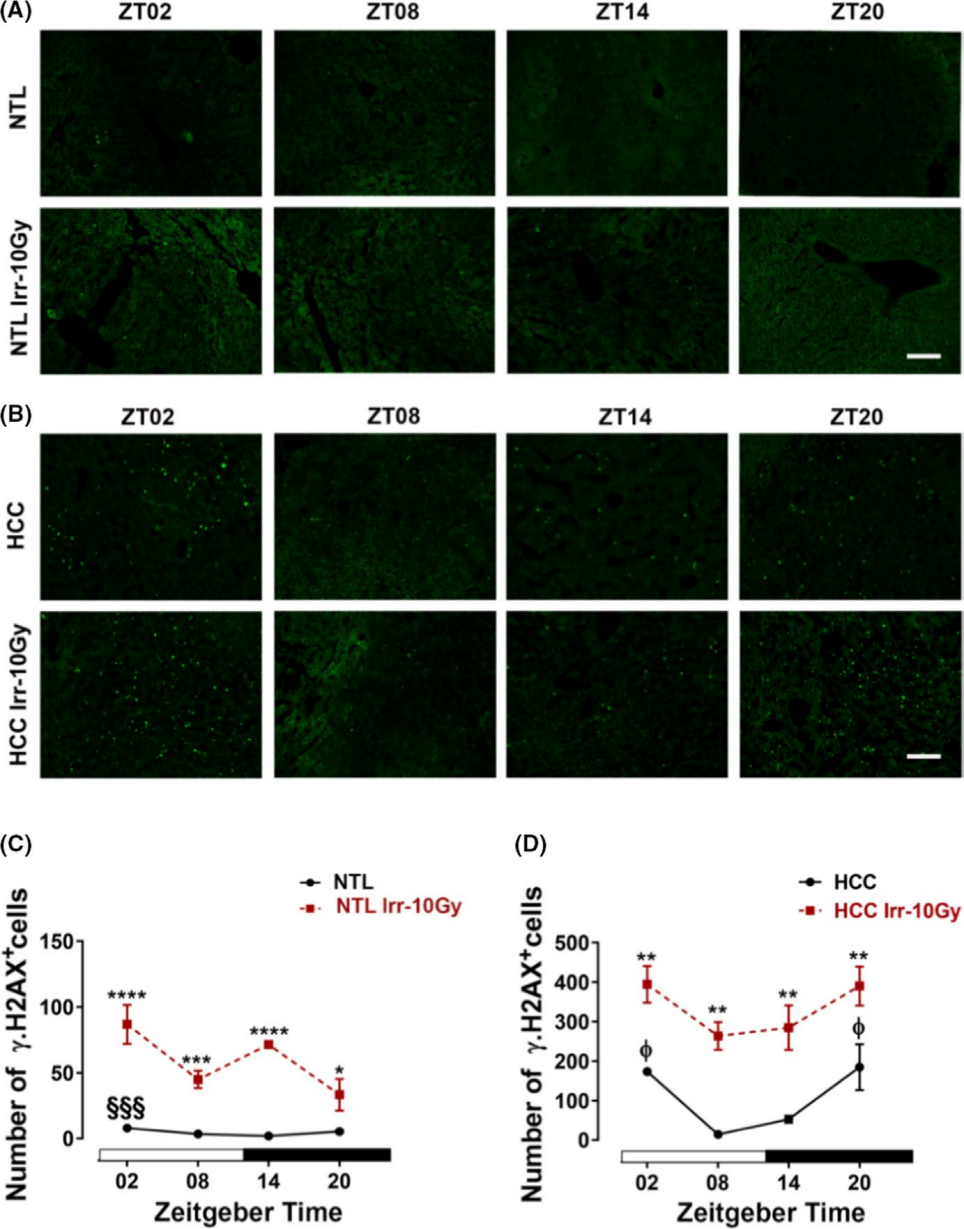
γ‐H2AX in ex vivo samples of hepatocellular carcinoma (HCC) and non‐tumoral liver (NTL) with or without irradiation. At different *Zeitgeber* times (ZT00= the onset of the light phase), mice were irradiated (Irr‐10 Gy) (*n* = 3/time point) or handled similarly but not irradiated. Forty‐eight hours later, mice were sacrificed at the same ZTs. Representative photomicrographs of γ‐H2AX immunoreaction in NTL (A) and HCC (B). Quantification of γ‐H2AX immunoreactive (+) cells in NTL (C) and HCC (D). Plotted are the mean numbers ± SEM of immunoreactive (+) cells. White and black bars indicate the light and dark phases, respectively. §§§: *p *< 0.001 differences between this ZT and ZT14. φ: *p *< 0.05 differences between this ZT and ZT08. *: *p *< 0.05; **: *p *< 0.01; ***: *p *< 0.001; ****: *p *< 0.0001 differences between the nonirradiated and irradiated group. Scale bars, 100 μm

In nonirradiated NTL, the number of γ‐H2AX+ cells showed a peak in the early light phase (ZT02) which was significantly different from the trough at the early dark phase (ZT14) (*p *< 0.0001, Figure 2A,C). In nonirradiated HCC, the number of γ‐H2AX+ cells was significantly higher at ZT02 and ZT20 (*p *< 0.0001) as compared with NTL. The HCC revealed two peaks, one at ZT02 and a second at ZT20. The two peaks were significantly different from the minimum at ZT08 (*p *< 0.05, Figure 2D).

Irradiation led to an increase in the number of γ‐H2AX+ cells at all four ZTs in HCC and NTL as compared with nonirradiated samples. As compared with nonirradiated NTL, the number of γ‐H2AX+ cells in NTL was higher when the animals were irradiated at ZT02 and ZT14 (90.7% and 80%, respectively; *p *< 0.0001) than at ZT08 (47.9%, *p *< 0.001) and ZT20 (32.2%, *p *< 0.05; Figure 2A,C and Figure S6). In irradiated HCC, the number of γ‐H2AX+ cells was significantly increased (*p *< 0.01) as compared with the nonirradiated HCC at all ZTs (Figure 2B,D).

### 3.2 Clock gene expression in HCC and NTL in mice without and with irradiation (10 Gy) at four different ZTs (ex vivo)

**FIGURE 3:**
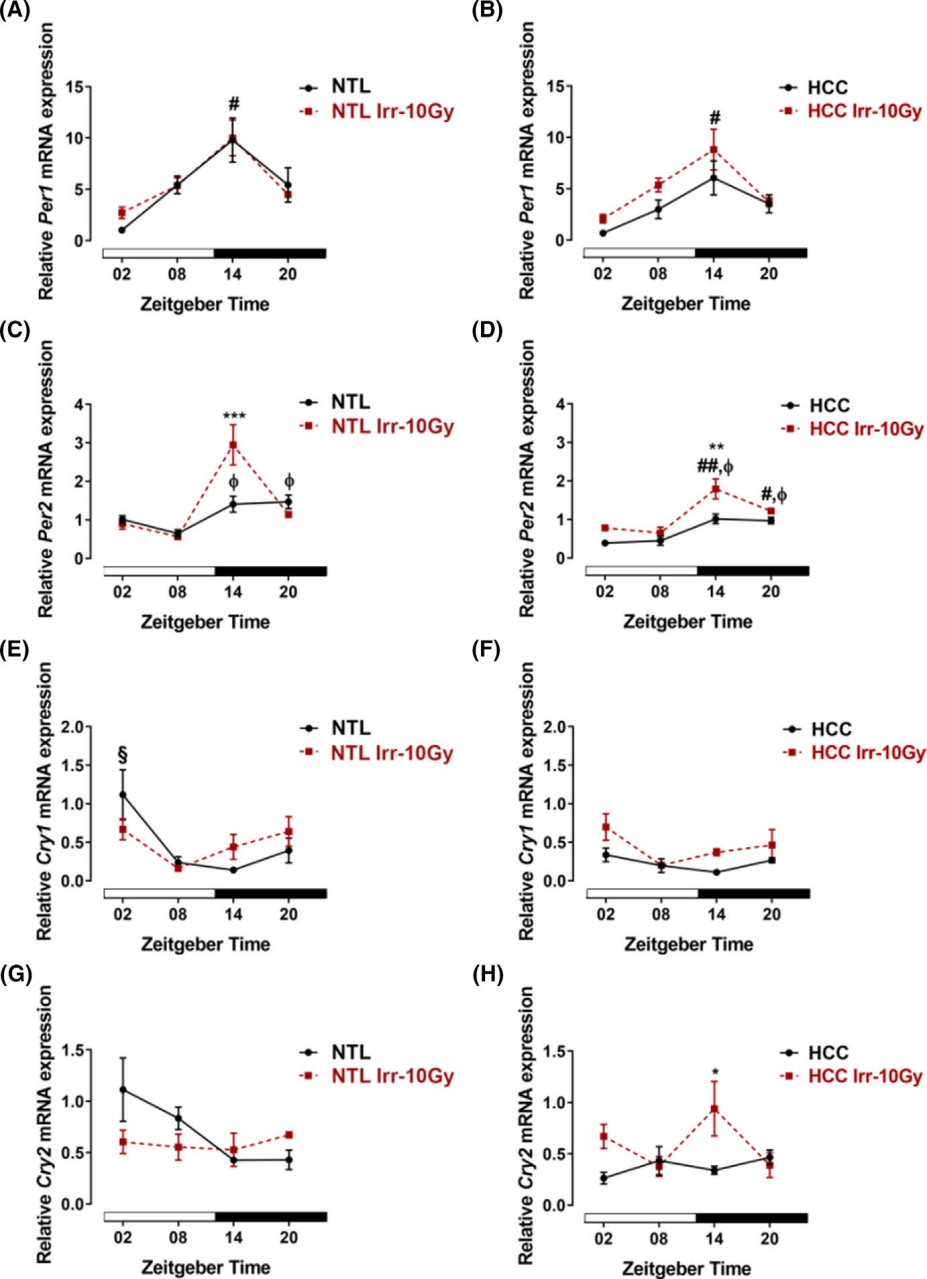
Clock gene expressions in ex vivo samples of hepatocellular carcinoma (HCC, left panel) and surrounding non‐tumoral liver (NTL, right panel) without or with irradiation (Irr‐10 Gy). At different *Zeitgeber* times (ZT00 = the onset of the light phase), mice were irradiated (*n* = 3/time point) or handled similarly but not irradiated. Forty‐eight hours later, mice were sacrificed at the same ZTs. Relative expression of *Per1* in NTL (A) and HCC (B). Relative expression of *Per2* in NTL (C) and HCC (D). Relative expression of *Cry1* in NTL (E) and HCC (F). Relative expression of *Cry2* in NTL (G) and HCC (H). Plotted are the mean relative mRNA expressions ± SEM. White and black bars indicate the light and dark phases, respectively. #: *p *< 0.05; ##: *p *< 0.01 differences between this ZT and ZT02. φ: *p *< 0.05 differences between this ZT and ZT08. §: *p *< 0.05 differences between this ZT and ZT14. *: *p *< 0.05; **: *p *< 0.01; ***: *p *< 0.001 differences between the nonirradiated and irradiated group

The relative expression of *Per1* in nonirradiated NTL and HCC showed a peak at ZT14 which was significantly different from the value at ZT02 (*p *< 0.05). There were no differences between HCC and NTL among ZTs (*p *> 0.05). The relative expression of *Per1* did not differ when the irradiated NTL and HCC were compared with the nonirradiated samples at all ZTs (*p *> 0.05, Figure 3A,B).

The relative expression of *Per2* in nonirradiated NTL and HCC was higher at ZT14 and ZT20 as compared with ZT08 (*p *< 0.05). At ZT02, the relative expression of *Per2* was lower in HCC than in NTL (*p *< 0.05). When the mice were irradiated at ZT14, the relative expression of *Per2* was significantly increased in both the NTL and the HCC (*p *< 0.001; *p *< 0.01, Figure 3C,D).

The relative expression of *Cry1* showed a peak at ZT02 in nonirradiated NTL which was significantly different from the value at ZT14 (*p *< 0.05). In nonirradiated HCC, the relative expression of *Cry1* was not different among the ZTs and was significantly lower as compared with nonirradiated NTL at ZT02 (*p *< 0.01). Irradiation had no effect on the relative expression of *Cry1* in HCC or NTL (*p *> 0.05, Figure 3E,F).

The relative expression of *Cry2* had a peak at ZT02 in nonirradiated NTL which showed a tendency to be significantly different from the value at ZT20 (*p* = 0.08). In nonirradiated HCC, the relative expression of *Cry2* was not different among the ZTs. At ZT02, the relative expression of *Cry2* was lower in HCC than in NTL (*p *< 0.01). When the mice were irradiated at ZT14, the relative expression of *Cry2* was increased in HCC as compared with the nonirradiated HCC (*p *< 0.05, Figure 3G,H).

**FIGURE 4:**
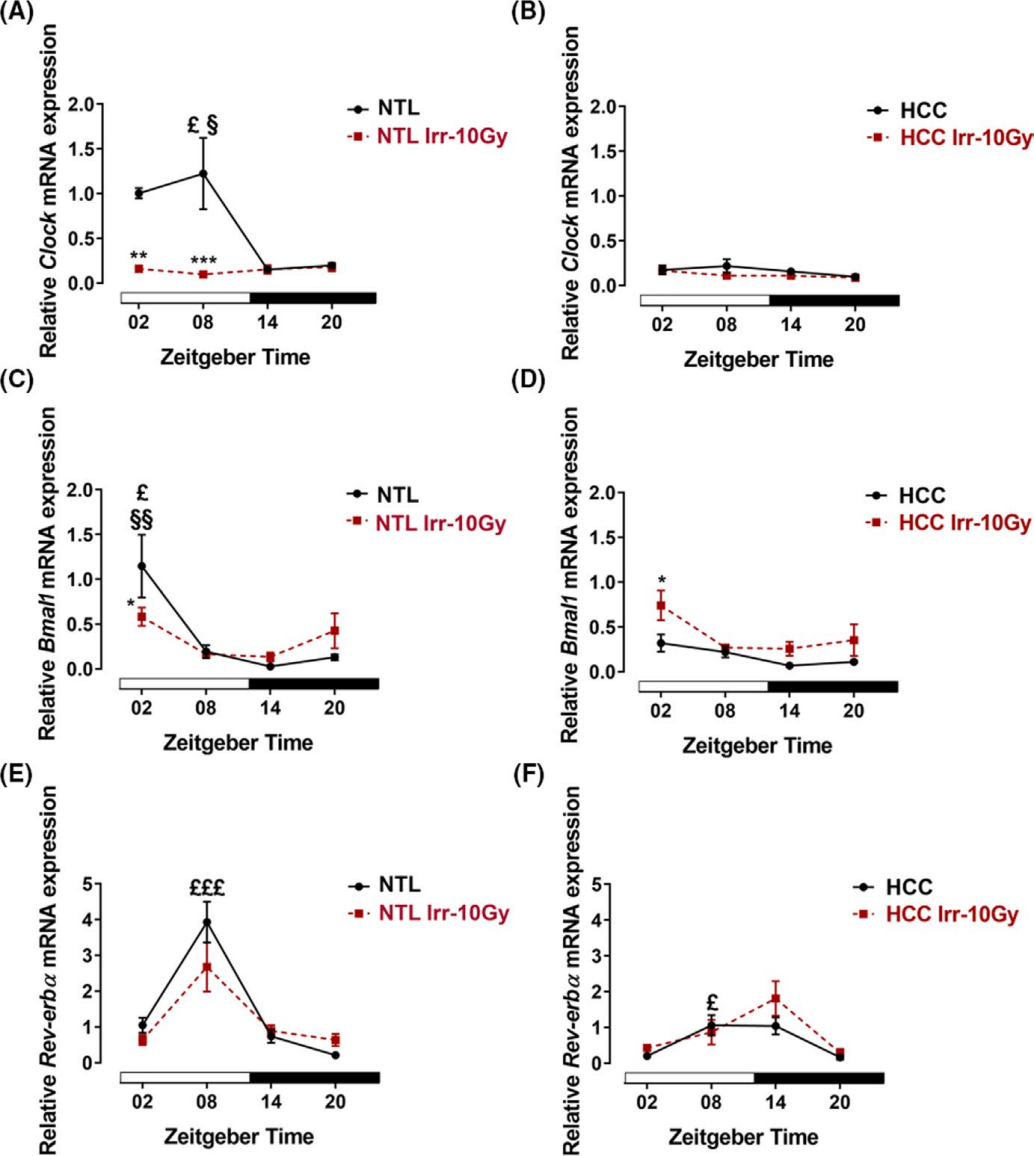
Clock gene expressions in ex vivo samples of hepatocellular carcinoma (HCC, left panel) and non‐tumoral liver (NTL, right panel) without or with irradiation (Irr‐10 Gy). At different *Zeitgeber* times (ZT00 = the onset of the light phase), mice were irradiated (*n* = 3/time point) or handled similarly but not irradiated. Forty‐eight hours later, mice were sacrificed at the same ZTs. Relative expression of *Clock* in NTL (A) and HCC (B). Relative expression of *Bmal1* in NTL (C) and HCC (D). Relative expression of *Rev*‐*erbα* in NTL (E) and HCC (F). Plotted are the mean relative mRNA expressions ± SEM. White and black bars indicate the light and dark phases, respectively. §: *p *< 0.05; §§: *p *< 0.01 differences between this ZT and ZT14. £: *p *< 0.05; £££: *p *< 0.001 differences between this ZT and ZT20. *: *p *< 0.05; **: *p *< 0.01; ***: *p *< 0.001 differences between the nonirradiated and irradiated group

The relative expression of *Clock* in nonirradiated NTL showed a peak at ZT08 which was significantly different from ZT14 to ZT20 (*p *< 0.05). In nonirradiated HCC, the relative expression of *Clock* was not different among the ZTs (*p *> 0.05) and was lower at ZT02 (*p* < 0.01) and ZT08 (*p* < 0.001) as compared with NTL. In NTL irradiated at ZT02 (*p *< 0.01) and ZT08 (*p *< 0.001), the relative expression of *Clock* was reduced as compared with the nonirradiated NTL (Figure 4A,B).

The relative expression of *Bmal1* in nonirradiated NTL showed a peak at ZT02 which was significantly different from ZT14 (*p *< 0.01) and ZT20 (*p *< 0.05). In nonirradiated HCC, the relative expression of *Bmal1* was not different among the ZTs (*p *> 0.05) and was lower at ZT02 as compared with NTL (*p *< 0.01). After irradiation at ZT02, the relative expression of *Bmal1* was decreased in irradiated NTL and increased in HCC as compared with nonirradiated samples (*p *< 0.05, Figure 4C,D).

The relative expression of *Rev*‐*erbα* showed a peak at (ZT08) in nonirradiated NTL (*p *< 0.001) and HCC (*p *< 0.05) which differed from ZT20. At ZT08, the relative expression of *Rev*‐*erbα* was lower in HCC than in NTL (*p *< 0.0001). Irradiation of the mice had no effect on the relative expression of *Rev*‐*erbα* (*p *> 0.05, Figure 4E,F).

The effects of irradiation on NTL and HCC are summarized in Table 2.

**TABLE 2:** Effects of irradiation on NTL and HCC. Statistical analysis by means of Sidak's multiple comparisons test

Time point	Mean NTL ± SE	Mean HCC ± SE	Significance
Ki67			
ZT02	3.92 ± 0.91	30.99 ± 8.18	*p* = 0.0001^***^
ZT08	2.72 ± 0.16	11.85 ± 3.73	*p* = 0.2569
ZT14	2.96 ± 0.29	11.65 ± 2.42	*p* = 0.2979
ZT20	2.94 ± 0.4	11.55 ± 1.31	*p* = 0.3072
γ‐H2AX			
ZT02	86.95 ± 14.79	395.0 ± 46.42	*p* < 0.0001^****^
ZT08	45.11 ± 6.56	264.3 ± 35.05	*p* = 0.0014^**^
ZT14	71.54 ± 1.24	285.4 ± 56.32	*p* = 0.0017^**^
ZT20	33.52 ± 12	390.9 ± 49.32	*p* < 0.0001^****^
*Per1*			
ZT02	2.71 ± 0.56	2.08 ± 0.42	*p* = 0.9783
ZT08	5.35 ± 0.77	5.36 ± 0.66	*p* > 0.9999
ZT14	9.997 ± 1.73	8.80 ± 1.99	*p* = 0.8810
ZT20	4.49 ± 0.14	3.69 ± 0.43	*p* = 0.9699
*Per2*			
ZT02	0.90 ± 0.14	0.78 ± 0.06	*p* = 0.9847
ZT08	0.56 ± 0.04	0.65 ± 0.15	*p* = 0.9973
ZT14	2.95 ± 0.52	1.79 ± 0.26	*p* = 0.0053^**^
ZT20	1.14 ± 0.07	1.22 ± 0.05	*p* = 0.9985
*Cry1*			
ZT02	0.67 ± 0.13	0.67 ± 0.17	*p* = 0.9997
ZT08	0.16 ± 0.03	0.20 ± 0.02	*p* = 0.9995
ZT14	0.44 ± 0.16	0.37 ± 0.04	*p* = 0.9958
ZT20	0.64 ± 0.19	0.46 ± 0.2	*p* = 0.8832
*Cry2*			
ZT02	0.60 ± 0.11	0.67 ± 0.12	*p* = 0.9938
ZT08	0.55 ± 0.13	0.38 ± 0.09	*p* = 0.8719
ZT14	0.53 ± 0.16	0.94 ± 0.26	*p* = 0.2184
ZT20	0.67 ± 0.03	0.39 ± 0.12	*p* = 0.5594
*Clock*			
ZT02	0.16 ± 0.03	0.16 ± 0.03	*p* > 0.9999
ZT08	0.1 ± 0.01	0.11 ± 0.02	*p* = 0.9980
ZT14	0.15 ± 0.05	0.11 ± 0.02	*p* = 0.8575
ZT20	0.18 ± 0.05	0.09 ± 0.03	*p* = 0.2566
*Bmal1*			
ZT02	0.58 ± 0.10	0.74 ± 0.16	*p* = 0.8093
ZT08	0.16 ± 0.03	0.27 ± 0.03	*p* = 0.9664
ZT14	0.13 ± 0.05	0.26 ± 0.08	*p* = 0.9442
ZT20	0.43 ± 0.19	0.35 ± 0.17	*p* = 0.9915
*Rev‐erbα*			
ZT02	0.64 ± 0.14	0.43 ± 0.03	*p* = 0.9755
ZT08	2.68 ± 0.69	0.87 ± 0.34	*p* = 0.0035^**^
ZT14	0.89 ± 0.16	1.81 ± 0.48	*p* = 0.2182
ZT20	0.64 ± 0.17	0.29 ± 0.11	*p* = 0.9120

### 3.3 Blood cell counts in mice without and with irradiation (10 Gy) at four different ZTs (ex vivo)

**FIGURE 5:**
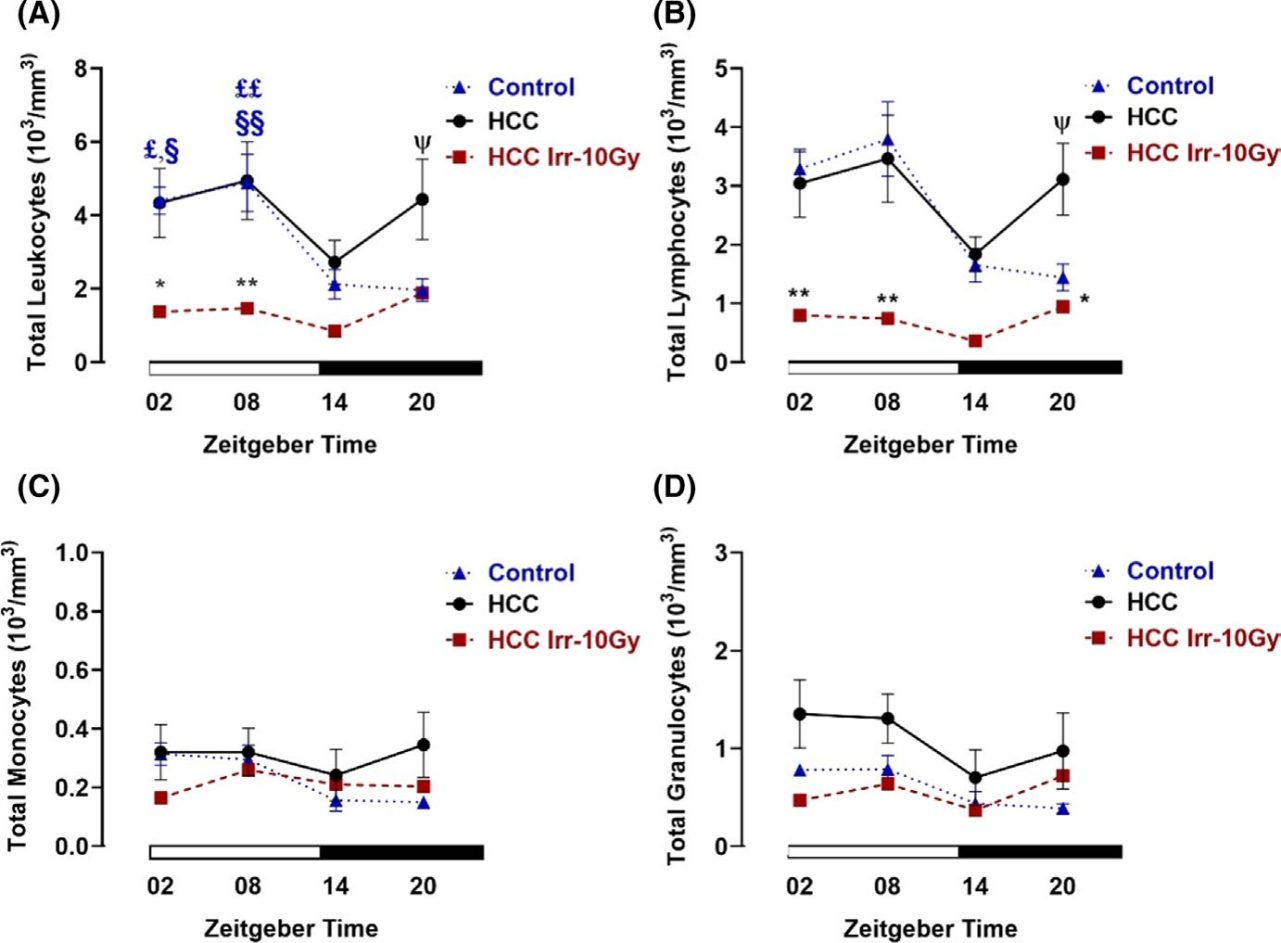
Blood cell analysis in control and hepatocellular carcinoma (HCC)‐bearing mice without and with irradiation. At different *Zeitgeber* times (ZT00 = the onset of the light phase), mice were irradiated (Irr) with a dose of 10 Gy (*n* = 3–6/time point) or handled similarly but not irradiated. Forty‐eight hours later, mice were sacrificed and the blood was collected at the same ZTs. (A) Total leukocyte numbers. (B) Lymphocyte numbers. (C) Monocyte numbers. (D) Granulocyte numbers. Plotted are the mean numbers ± SEM. White and black bars indicate the light and dark phases, respectively. §: *p *< 0.05; §§: *p *< 0.01 differences between this ZT and ZT14. £: *p *< 0.05; ££: *p *< 0.01 differences between this ZT and ZT20. *: *p *< 0.05; **: *p *< 0.01 differences between nonirradiated and irradiated animals. ψ: *p *< 0.05 differences between control and nonirradiated animals

Control mice showed a daily variation in the total number of leukocytes with higher levels during the light phase than during the dark phase. In the nonirradiated HCC group, there was no significant daily variation of leukocytes. At ZT20, the leukocytes and lymphocytes number were significantly higher as compared with the control group (*p *< 0.05). In the irradiated group, the number of leukocytes at ZT02 (*p *< 0.05) and ZT08 (*p *< 0.01) was significantly decreased. In addition, irradiation caused a highly significant decrease at ZT02 and ZT08 (*p *< 0.01), a significant decrease at ZT20 (*p *< 0.05) and nonsignificant decrease at ZT14 in the total lymphocytes number as compared with the nonirradiated HCC mice (Figure 5A,B).

There were no differences in the total number of monocytes and granulocytes in both the control group and the HCC mice. After irradiation, no differences were observed in their numbers regardless of the time point of irradiation as compared with the nonirradiated HCC mice (Figure 5C,D). There were no differences in the number of erythrocytes, platelets and the hemoglobin concentration in control, nonirradiated, and irradiated mice (Figure S7A–C).

## 4 DISCUSSION

This study addresses the question whether chronotherapy may be beneficial for radiotherapy of HCC in a DEN‐induced HCC mouse model, which closely mimics human HCC, in view of the multistage development of the liver tumors over months prior for them to become established and to progress. Prior to radiotherapy of whole animals, in vitro experiments were performed to determine the efficient dose (10 Gy).

### 4.1 Proliferation and DNA damage in nonirradiated animals

As expected, proliferation was much higher in HCC than in NLT and the daily variation differed between HCC and NLT. The number of Ki67+ cells showed one peak at ZT02 in NTL, while two peaks were found in HCC: a maximum at the late activity phase (ZT20) and a second peak in the early inactivity phase (ZT02). Two proliferation peaks were also observed in HCC of double transgenic c‐myc/TGFα^11^ and this seems to be a characteristic feature of fast‐growing tumors.^27^ The daily variations of cell proliferation in HCC and the NTL result from the circadian oscillation of the cell cycle molecules which either promote or inhibit cell cycle proliferation (e.g., CycD1 and c‐Myc).^28^, ^29^

DNA damage also showed a daily variation in NTL and HCC. In NTL, the number of γ‐H2AX+ cells showed only one peak at ZT02 while in HCC they had an additional peak at ZT20. The increase of γ‐H2AX+ cells in HCC during the second half of the dark phase is consistent with previous findings.^11^

### 4.2 Proliferation and DNA damage in irradiated animals

Irradiation inhibited proliferation in NTL during the light phase, but had little effects during the dark phase. In HCC, irradiation inhibited proliferation at all time points with the strongest effect observed at ZT20. Importantly, the effect of irradiation was most pronounced when applied at the proliferation peaks which were different between the HCC and NTL. Notably, irradiation at ZT20 had a low antiproliferative effect on NTL but had the highest antiproliferative effect on the HCC. With regard to DNA damage, irradiation led to an increase in the number of γ‐H2AX+ cells at all four ZTs in HCC and NTL as compared with nonirradiated samples. In NTL, the number of γ‐H2AX+ cells in NTL was higher at ZT02 and ZT14 than at ZT08 and ZT20. In HCC, irradiation caused an increase in the number of γ‐H2AX+ cells at all ZTs. The increase in the number of γ‐H2AX+ cells after irradiation confirms that the irradiation induces the DNA‐DSBs repairing mechanism and contributes to the γ‐H2AX response.^17^, ^30^ Importantly, radiotherapy treatment during the late activity phase (ZT20) had the lowest effect on DNA‐DSBs damage in NTL and caused effective damage in HCC. From these data it can be inferred that the late activity phase is the optimal time point for radiotherapy of DEN‐induced HCC in mice.

### 4.3 Effects on blood cells of irradiated animals

As expected irradiation did not affect hemoglobin concentration and erythrocyte numbers since erythrocytes circulate for 120 days in the blood stream and a reduction of their numbers is a long‐term side effect of irradiation.

Irradiation caused a significant reduction in the total number of leukocytes and lymphocytes which conforms to data on white blood cell counts in patients^31^ and the observation that antimitotic therapy led to a dramatic decrease in the number of circulating lymphocytes independent of functional molecular clockwork.^32^ The reduction in lymphocyte numbers may reflect an enhanced lymphocyte apoptosis due to increased glucocorticoid levels recently demonstrated in irradiated mice with HCC.^33^ Notably, irradiation at ZT14 and ZT20 had no significant effect on the total leukocyte numbers. Thus, these time points might be preferable for radiotherapy in terms of reducing this severe side effect.

### 4.4 Expression of clock genes in NLT and HCC of nonirradiated animals

Disruption or mutation of clock genes is associated with genomic instability and increased proliferation rate, both favorable conditions for carcinogenesis.^2^, ^22^ Thus, we analyzed the expression of seven core clock genes in HCC and NTL. In NTL, all clock genes showed a time‐dependent variation consistent with previous observations.^11^ In HCC, expression of clock genes, *Cry1*, *Cry2*, *Clock*, and *Bmal1* showed a time‐dependent decrease as compared with NTL. Clock gene expression is differently altered in various tumors^34^, ^35^, ^36^, ^37^ for multiple reasons. One major reason is a hypoxia and the hypoxia transcription factors (e.g., HIF‐1α and HIF‐1β) with many consequences such as a lack of access to circadian resetting cues in the blood and inhibition of the transcription of clock genes.^38^, ^39^ In addition, hyper‐methylation of clock gene promoter regions is discussed as possible reason for clock gene dysregulation in tumors.^37^, ^39^, ^40^

### 4.5 Expression of clock genes in NLT and HCC of irradiated animals

To date, little is known about the effects of radiotherapy on the molecular clockwork which controls several rhythmic cell functions and thus affecting tolerability and efficacy of anticancer treatments.^41^, ^42^ Mice with mutations/deletion in the clock genes, *Clock*/*Bmal1* and *Per1*/*2*, showed enhanced chemotherapy‐ or gamma radiation‐induced toxicity in the healthy tissue.^32^, ^43^ An effect of gamma irradiation on clock gene expression in liver has been reported before in OSC and with the whole animals.^13^, ^29^ Most remarkably, the clock gene expression pattern in the tumor predicts the response of tumor patients to chemo‐radiotherapy,^42^ emphasizing the role of the molecular clockwork in the efficacy of cancer treatment. Our results showed that irradiation resulted in a downregulation in expression of *Clock* (ZT02,08) and *Bmal1* (ZT02) and an upregulation in expression of *Per2* (ZT14) in NLT. Importantly, irradiation at ZT20 had no effect on clock gene expression in NTL. This is consistent with a low impact of irradiation on proliferation and DNA‐DSBs in NTL at this time point. In turn, DNA damages which followed the ionizing radiation may induce phase shifting of the molecular clockwork as shown in Rat‐1 fibroblasts.^44^ This indicates the close connection between DNA damage/repair mechanism and circadian clock and may explain the dysregulation of the molecular clockwork in NTL after irradiation at all ZTs except ZT20 which had the lowest effect on DNA‐DSBs.

## 5 CONCLUSION AND TRANSLATIONAL ASPECTS

All results of this study on DEN‐induced HCC in mice indicate the late activity phase as optimal time point for radiotherapy at which the ratio between efficacy of tumor treatment and toxic side effects was maximal. Other studies, however, reported that radiation tolerance assessed by body weight loss and bone marrow suppression, increased in rodents in the late inactivity phase.^45^, ^46^ Most likely, these differences are due to the tumor type and organ‐dependent radiation tolerance. Differences in times of sensitivity/resistance to irradiation were also observed in many clinical studies reviewed in.^47^, ^48^ Thus, morning radiotherapy tended to be best tolerated by the oral mucosa patients.^49^ Patients with breast cancer who received radiotherapy early morning showed less skin reaction as compared with the group who received the radiotherapy late afternoon (after 3 PM).^50^ On the other hand, patients with prostate adenocarcinoma showed less side effects (GI complications) when the radiotherapy was applied in the late activity phase (before 5 PM) as compared with the early inactivity phase (after 5 PM)^51^ and patients with cervical carcinoma revealed less intestinal mucositis when the radiotherapy was applied after 6 PM as compared with the morning group.^52^

An important question is whether the data of this study are helpful to determine the optimal time point for radiotherapy of HCC in humans. Time points which are determined in nocturnal species (mouse) might be easily transferred to diurnal species (e.g., human), since chronotherapeutic strategies and antimitotic therapies seem to have the same phase relation with the rest‐activity cycle in mice and humans.^41^, ^53^, ^54^, ^55^ We defined the late activity phase as the optimal time point to apply the radiotherapy for HCC‐bearing mice and future clinical studies should clarify whether the late activity phase is the optimal time point to apply the radiotherapy for HCC patients. These studies should also consider that in humans, the organization of the circadian system has interindividual differences resulting in different chronotypes.

## CONFLICTS OF INTEREST

The authors have no conflict of interest.

## POSTED ON PREPRINT SERVERS

A working version manuscript was posted on preprint servers under the following link: https://www.preprints.org/manuscript/202101.0603/v1 10.20944/preprints202101.0603.v1.

## ETHICS STATEMENT

Animals were used according to accepted standards of humane animal care and federal guidelines and Directive 2010/63/EU of the European Union. All experiments were approved by the Regierungspräsidium Darmstadt and the Landesamt für Natur, Umwelt und Verbraucherschutz NRW (Reference number: AZ 81‐02.04.2018‐A146).

## Supporting information

Supplementary Material

## Data Availability

Data will be made available from the corresponding author upon reasonable request.

## References

[1] IARC . Latest Global Cancer Data: Cancer Burden Rises to 18.1 Million New Cases and 9.6 Million Cancer Deaths in 2018. PRESS RELEASE. 2018;N° 263. IARC.

[2] Sánchez DI , González‐Fernández B , Crespo I , et al. Melatonin modulates dysregulated circadian clocks in mice with diethylnitrosamine‐induced hepatocellular carcinoma. J Pineal Res. 2018;65(3):e12506. 29770483 10.1111/jpi.12506

[3] Sangro B , Sarobe P , Hervás‐Stubbs S , Melero I . Advances in immunotherapy for hepatocellular carcinoma. Nat Rev Gastroenterol Hepatol. 2021;18(8):525–543. 33850328 10.1038/s41575-021-00438-0 PMC8042636

[4] Prieto J , Melero I , Sangro B . Immunological landscape and immunotherapy of hepatocellular carcinoma. Nat Rev Gastroenterol Hepatol. 2015;12(12):681‐700. 26484443 10.1038/nrgastro.2015.173

[5] Bang A , Dawson LA . Radiotherapy for HCC: ready for prime time? JHEP Rep. 2019;1(2):131‐137. 32039361 10.1016/j.jhepr.2019.05.004 PMC7001576

[6] Kutlu R , Karatoprak S . Radioembolization for hepatocellular carcinoma in downstaging and bridging for liver transplantation. J Gastrointest Cancer. 2020;51(4):1157‐1164. 32880041 10.1007/s12029-020-00492-y

[7] Lee YH , Tai D , Yip C , Choo SP , Chew V . Combinational immunotherapy for hepatocellular carcinoma: radiotherapy, immune checkpoint blockade and beyond. Front Immunol. 2020;11:568759. 33117354 10.3389/fimmu.2020.568759 PMC7561368

[8] Wild AT , Gandhi N , Chettiar ST , et al. Concurrent versus sequential sorafenib therapy in combination with radiation for hepatocellular carcinoma. PLoS One. 2013;8(6):e65726. 23762417 10.1371/journal.pone.0065726 PMC3675179

[9] Ohri N , Dawson LA , Krishnan S , et al. Radiotherapy for hepatocellular carcinoma: new indications and directions for future study. J Natl Cancer Inst. 2016;108(9):doi:10.1093/jnci/djw133 PMC6279296 27377923

[10] Chen CP . Role of radiotherapy in the treatment of hepatocellular carcinoma. J Clin Transl Hepatol. 2019;7(2):183‐190. 31293919 10.14218/JCTH.2018.00060 PMC6609847

[11] Hassan SA , Schmithals C , von Harten M , Piiper A , Korf H‐W , von Gall C . Time‐dependent changes in proliferation, DNA damage and clock gene expression in hepatocellular carcinoma and healthy liver of a transgenic mouse model. Int J Cancer. 2021;148:226‐237. 32700769 10.1002/ijc.33228

[12] Harper E , Talbot CJ . Is it time to change radiotherapy: the dawning of chronoradiotherapy? Clin Oncol (R Coll Radiol). 2019;31(5):326‐335. 30902558 10.1016/j.clon.2019.02.010

[13] Muller MH , Rodel F , Rub U , Korf HW . Irradiation with X‐rays phase‐advances the molecular clockwork in liver, adrenal gland and pancreas. Chronobiol Int. 2015;32:27‐36. 25140390 10.3109/07420528.2014.949735

[14] Johnson K , Chang‐Claude J , Critchley A‐M , et al. Genetic variants predict optimal timing of radiotherapy to reduce side‐effects in breast cancer patients. Clin Oncol (R Coll Radiol). 2019;31(1):9‐16. 30389261 10.1016/j.clon.2018.10.001

[15] Rebouissou S , La Bella T , Rekik S , et al. Proliferation markers are associated with MET expression in hepatocellular carcinoma and predict tivantinib sensitivity in vitro. Clin Cancer Res. 2017;23(15):4364‐4375. 28246274 10.1158/1078-0432.CCR-16-3118

[16] Rahn DA , Ray DK , Schlesinger DJ , et al. Gamma knife radiosurgery for brain metastasis of nonsmall cell lung cancer: is there a difference in outcome between morning and afternoon treatment? Cancer. 2011;117(2):414‐420. 20830691 10.1002/cncr.25423

[17] Kuo LJ , Yang L . γ‐H2AX – a novel biomarker for DNA double‐strand breaks. In vivo. 2008;22:305‐310. 18610740

[18] Wood PA , Du‐Quiton J , You S , Hrushesky WJ . Circadian clock coordinates cancer cell cycle progression, thymidylate synthase, and 5‐fluorouracil therapeutic index. Mol Cancer Ther. 2006;5(8):2023‐2033. 16928823 10.1158/1535-7163.MCT-06-0177

[19] Zhou D , Wang Y , Chen LU , et al. Evolving roles of circadian rhythms in liver homeostasis and pathology. Oncotarget. 2016;7(8):8625‐8639. 26843619 10.18632/oncotarget.7065 PMC4890992

[20] Schibler U , Ripperger J , Brown SA . Peripheral circadian oscillators in mammals: time and food. J Biol Rhythms. 2003;18(3):250‐260. 12828282 10.1177/0748730403018003007

[21] Korf H‐W , von Gall C . Circadian physiology. In: Pfaff DW , ed. Neuroscience in the 21st Century: From Basic to Clinical. Springer, New York; 2013:1813‐1845.

[22] Huisman SA , Oklejewicz M , Ahmadi AR , et al. Colorectal liver metastases with a disrupted circadian rhythm phase shift the peripheral clock in liver and kidney. Int J Cancer. 2015;136(5):1024‐1032. 25045881 10.1002/ijc.29089

[23] Yang FE , Vaida F , Ignacio L , et al. Analysis of weekly complete blood counts in patients receiving standard fractionated partial body radiation therapy. Int J Radiat Oncol Biol Phys. 1995;33(3):617‐717. 7558950

[24] Wersal C , Keller A , Weiss C , et al. Long‐term changes in blood counts after intraoperative radiotherapy for breast cancer—single center experience and review of the literature. Transl Cancer Res. 2019;8:1882‐1903. 10.21037/tcr.2019.09.05 PMC8799206 35116939

[25] Verrill C , Davies J , Millward‐Sadler H , Sundstrom L , Sheron N . Organotypic liver culture in a fluid‐air interface using slices of neonatal rat and adult human tissue–a model of fibrosis in vitro. J Pharmacol Toxicol Methods. 2002;48(2):103‐110. 14565567 10.1016/S1056-8719(03)00042-X

[26] Pfaffl MW . Quantification strategies in real‐time PCR. In: Bustin SA , ed. A‐Z of Quantitative PCR. International University Line (IUL); 2004:87‐112.

[27] Echave Llanos JM , Nash RE . Mitotic circadian rhythm in a fast‐growing and a slow‐growing hepatoma: mitotic rhythm in hepatomas. J Natl Cancer Inst. 1970;44(3):581‐585. 11515426

[28] Yang X , Wood PA , Ansell CM , et al. The circadian clock gene Per1 suppresses cancer cell proliferation and tumor growth at specific times of day. Chronobiol Int. 2009;26(7):1323‐1339. 19916834 10.3109/07420520903431301

[29] Fu L , Pelicano H , Liu J , Huang P , Lee C . The circadian gene Period2 plays an important role in tumor suppression and DNA damage response in vivo. Cell. 2002;111(1):41‐50. 12372299 10.1016/s0092-8674(02)00961-3

[30] Wang JS , Wang HJ , Qian HL . Biological effects of radiation on cancer cells. Mil Med Res. 2018;5(1):20. 29958545 10.1186/s40779-018-0167-4 PMC6026344

[31] Stone HB , Coleman CN , Anscher MS , McBride WH . Effects of radiation on normal tissue: consequences and mechanisms. Lancet Oncol. 2003;4(9):529‐536. 12965273 10.1016/s1470-2045(03)01191-4

[32] Antoch MP , Kondratov RV , Takahashi JS . Circadian clock genes as modulators of sensitivity to genotoxic stress. Cell Cycle. 2005;4(7):901‐907. 15917646 10.4161/cc.4.7.1792 PMC3774065

[33] Hassan SA , Ali AAH , Yassine M , et al. Relationship between locomotor activity rhythm and corticosterone levels during HCC development, progression, and treatment in a mouse model. J Pineal Res. 2021;70:e12724. 33615553 10.1111/jpi.12724

[34] Huisman SA , Ahmadi AR , IJzermans JNM , Verhoef C , van der Horst GTJ , de Bruin RWF . Disruption of clock gene expression in human colorectal liver metastases. Tumour Biol. 2016;37(10):13973‐13981. 27492458 10.1007/s13277-016-5231-7 PMC5097083

[35] Mteyrek A , Filipski E , Guettier C , Okyar A , Levi F . Clock gene Per2 as a controller of liver carcinogenesis. Oncotarget. 2016;7(52):85832‐85847. 27494874 10.18632/oncotarget.11037 PMC5349878

[36] Oshima T , Takenoshita S , Akaike M , et al. Expression of circadian genes correlates with liver metastasis and outcomes in colorectal cancer. Oncol Rep. 2011;25(5):1439. 21380491 10.3892/or.2011.1207

[37] Lin Y‐M , Chang JH , Yeh K‐T , et al. Disturbance of circadian gene expression in hepatocellular carcinoma. Mol Carcinog. 2008;47(12):925‐933. 18444243 10.1002/mc.20446

[38] Hunyor I , Cook KM . Models of intermittent hypoxia and obstructive sleep apnea: molecular pathways and their contribution to cancer. Am J Physiol‐Reg I. 2018;315(4):R669‐R687. 10.1152/ajpregu.00036.2018 29995459

[39] Morgan M , Dvuchbabny S , Martinez C‐A , Kerr B , Cistulli PA , Cook KM . The cancer clock is (Not) ticking: links between circadian rhythms and cancer. Clocks & Sleep. 2019;1(4):435‐458. 33089179 10.3390/clockssleep1040034 PMC7445810

[40] Yu C , Yang SL , Fang X , Jiang JX , Sun CY , Huang T . Hypoxia disrupts the expression levels of circadian rhythm genes in hepatocellular carcinoma. Mol Med Rep. 2015;11(5):4002‐4008. 25591621 10.3892/mmr.2015.3199

[41] Mormont MC , Levi F . Cancer chronotherapy: Principles, applications, and perspectives. Cancer. 2003;97(1):155‐169. 12491517 10.1002/cncr.11040

[42] Lu H , Chu Q , Xie G , et al. Circadian gene expression predicts patient response to neoadjuvant chemoradiation therapy for rectal cancer. Int J Clin Exp Pathol. 2015;8(9):10985‐10994. 26617816 PMC4637631

[43] Dakup PP , Porter KI , Gajula RP , Goel PN , Cheng Z , Gaddameedhi S . The circadian clock protects against ionizing radiation‐induced cardiotoxicity. Faseb J. 2020;34(2):3347‐3358. 31919902 10.1096/fj.201901850RR PMC9677419

[44] Oklejewicz M , Destici E , Tamanini F , Hut RA , Janssens R , van der Horst GT . Phase resetting of the mammalian circadian clock by DNA damage. Curr Biol. 2008;18(4):286‐291. 18291650 10.1016/j.cub.2008.01.047

[45] Newsome‐Tabatabai R , Rushton PS . Daily variation in radiosensitivity of circulating blood cells and bone marrow cellularity of mice. Comp Biochem Physiol A Comp Physiol. 1984;78(4):779‐783. 6149053 10.1016/0300-9629(84)90633-9

[46] Haus E . Chronobiology of the mammalian response to ionizing radiation potential applications in oncology. Chronobiol Int. 2002;19(1):77‐100. 11962688 10.1081/cbi-120002592

[47] Bermúdez‐Guzmán L , Blanco‐Saborío A , Ramírez‐Zamora J , Lovo E . The time for chronotherapy in radiation oncology. Front Oncol. 2021;11: doi:10.3389/fonc.2021.687672 PMC8144648 34046365

[48] Shuboni‐Mulligan DD , Breton G , Smart D , Gilbert M , Armstrong TS . Radiation chronotherapy‐clinical impact of treatment time‐of‐day: a systematic review. J Neurooncol. 2019;145(3):415‐427. 31729636 10.1007/s11060-019-03332-7 PMC8130840

[49] Bjarnason GA , MacKenzie RG , Nabid A , et al. Comparison of toxicity associated with early morning versus late afternoon radiotherapy in patients with head‐and‐neck cancer: a prospective randomized trial of the National Cancer Institute of Canada Clinical Trials Group (HN3). Int J Radiat Oncol Biol Phys. 2009;73(1):166‐172. 18805649 10.1016/j.ijrobp.2008.07.009

[50] Noh JM , Choi DH , Park H , et al. Comparison of acute skin reaction following morning versus late afternoon radiotherapy in patients with breast cancer who have undergone curative surgical resection. J Radiat Res. 2014;55(3):553‐558. 24385471 10.1093/jrr/rrt141 PMC4014164

[51] Hsu F‐M , Hou W‐H , Huang C‐Y , et al. Differences in toxicity and outcome associated with circadian variations between patients undergoing daytime and evening radiotherapy for prostate adenocarcinoma. Chronobiol Int. 2016;33(2):210‐219. 26818960 10.3109/07420528.2015.1130049

[52] Shukla P , Gupta D , Bisht SS , et al. Circadian variation in radiation‐induced intestinal mucositis in patients with cervical carcinoma. Cancer. 2010;116(8):2031‐2035. 20162717 10.1002/cncr.24867

[53] Pritchett D , Reddy AB . Circadian clocks in the hematologic system. J Biol Rhythms. 2015;30(5):374‐388. 26163380 10.1177/0748730415592729

[54] Scheiermann C , Kunisaki Y , Frenette PS . Circadian control of the immune system. Nat Rev Immunol. 2013;13(3):190‐198. 23391992 10.1038/nri3386 PMC4090048

[55] Zoccoli G , Amici R . Sleep and autonomic nervous system. Curr Opin Physiol. 2020;15:128‐133.

