# Supplementary material for: Does timing matter in radiotherapy of hepatocellular carcinoma? An experimental study in mice
Source: Cancer Med. 2021 Sep 20;10(21):7712–25. doi: 10.1002/cam4.4277 (PMC8559477; doi:10.1002/cam4.4277)
Supplement: Supplementary file 1 — Supplementary Material [file CAM4-10-7712-s001.docx]

**Supplementary materials**


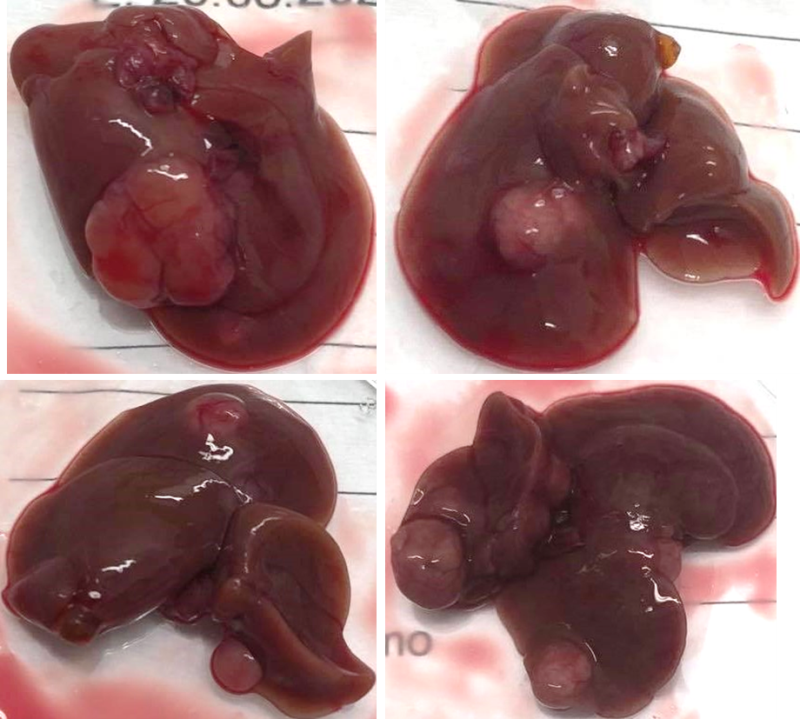


**Figure S1.** Representative photographs of single and multiple hepatocellular carcinomas (HCC) at the age of 7-10 months. The mice received a single injection of diethylnitrosamine (DEN) at the age of two weeks and chronic treatment of phenobarbital (PB) in the drinking water to accelerate the HCC induction.


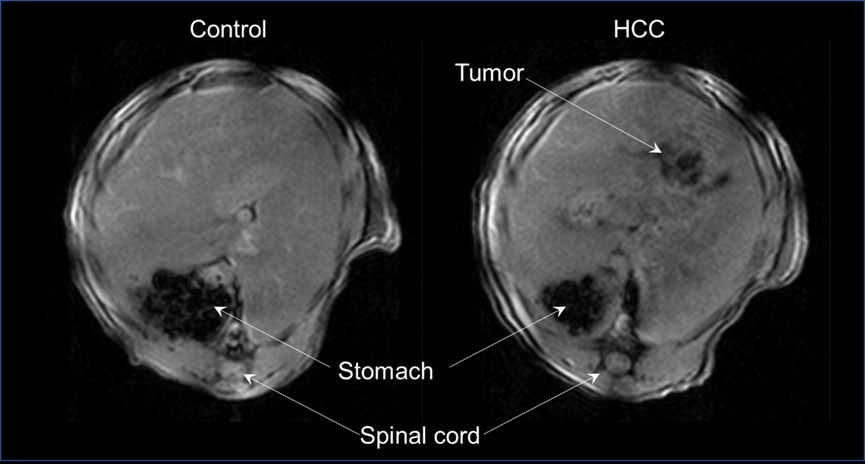


**Figure S2.** Representative axial MRI images from healthy and HCC bearing mice demonstrating the unequivocal identification of tumor tissue within the liver.


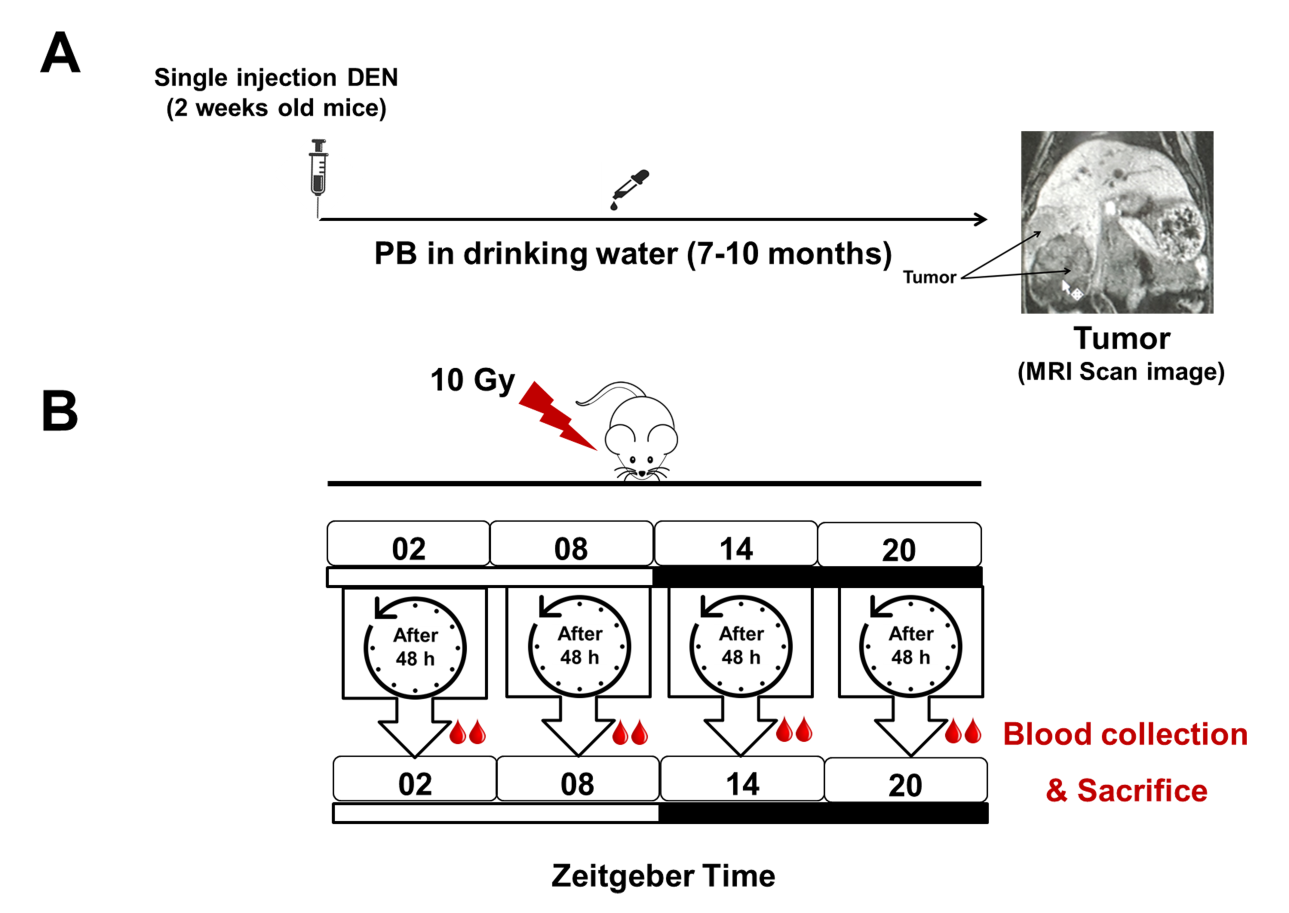


**Figure S3.** Diagram for the experimental design of tumor induction, animals' irradiation and *ex vivo* analyses. **A,** Transgenic *Per2::luc* mice (n=48 mice) received a single injection of diethylnitrosamine (DEN) at the age of two weeks and chronic treatment of phenobarbital (PB) in the drinking water to accelerate the hepatocellular carcinoma (HCC) induction. HCC developed in animals in either single or multiple tumors at the age of 7-10 months. Tumor development was screened via magnetic resonance imaging (MRI) and validated by post mortem inspection. **B,** 24 animals of the HCC bearing mice were selected for irradiation with a dose of 10 Gy at four different *Zeitgeber* time (ZT) points (ZT02, ZT08, ZT14 and ZT20) (6 animals per time point). 48 hours later, blood was collected and animals were sacrificed at the same ZTs used for irradiation. 12 animals (n=3/ZT) were perfused for immunohistochemistry and 12 animals (n=3/ZT) were used for real-time PCR by collecting and snap freezing the native tissue. White and black bars indicate the light and dark phases, respectively.

*Ki67 and γ-H2AX in OSCs of HCC and NTL without and with irradiation with two different doses at four different CTs (in vitro)*

In non-irradiated OSCs, the number of Ki67+ cells was very low and not different among the four CTs in NTL (*p> 0.05*, Fig. S4A, C). In HCC, the number of Ki67+ cells was significantly higher (*p< 0.0001*) as compared with NTL and showed a peak at CT02 which, however, did not differ from the values at the other CTs (*p> 0.05*, Fig. S4B, D).

Irradiation with 2 or 10 Gy had no effect on the number of Ki67+ cells at all CTs in NTL (*p> 0.05*, Fig. S4A, C). In HCC, irradiation with 2 Gy elicited no changes in the number of Ki67+ cells at any CT (*p> 0.05,* Fig. S4B, D). In contrast, after irradiation with a dose of 10 Gy at CT02, the number of Ki67+ cells was significantly lower as compared with the respective non-irradiated HCC (*p< 0.05*). Irradiation with 10 Gy had no effect on the number of Ki67+ cells at any other time point (Fig. S4B, D).


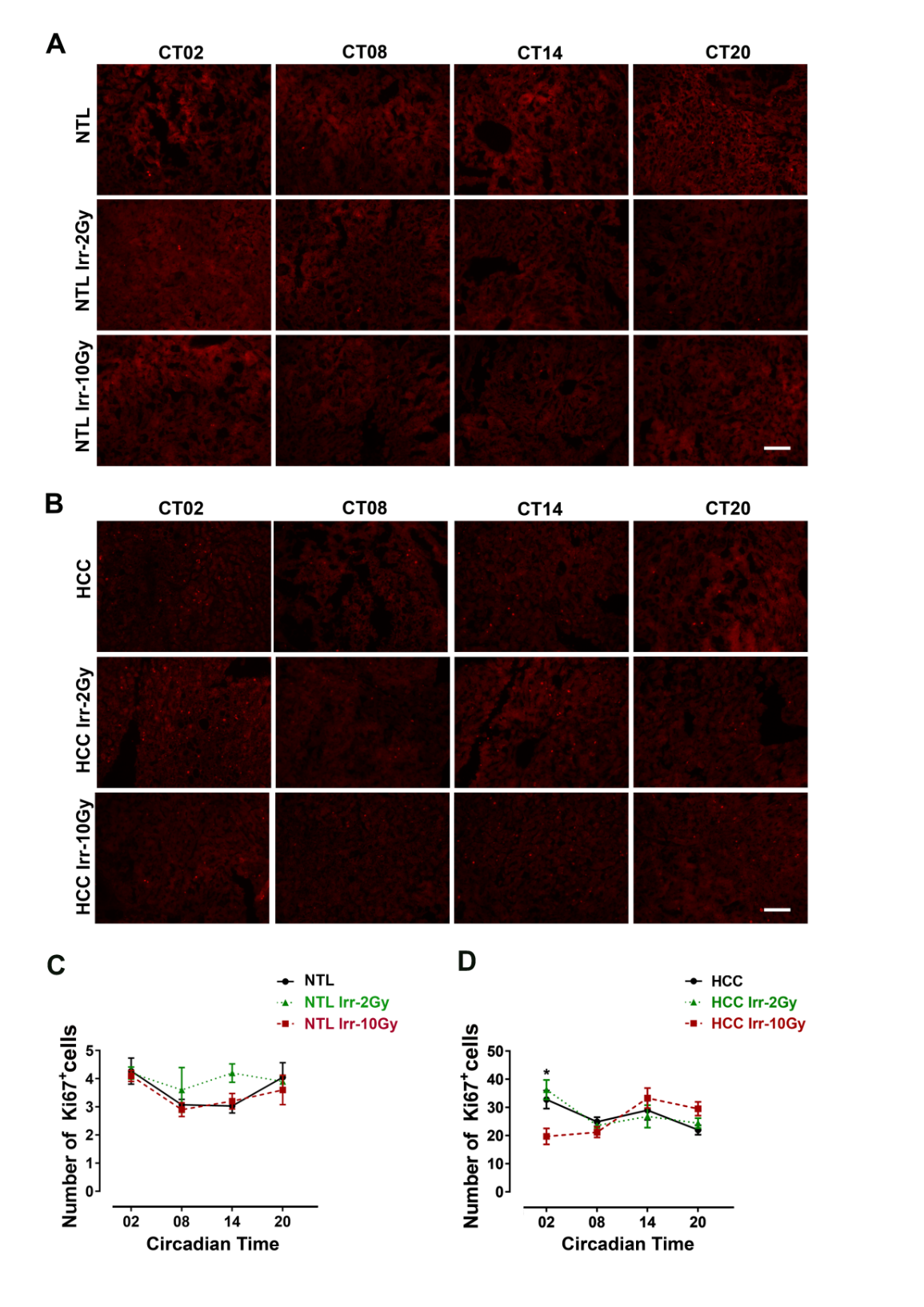


**Figure S4.** Ki67 in organotypic slice cultures (OSCs) of hepatocellular carcinoma (HCC) and surrounding non-tumoral liver (NTL) with or without irradiation. At different circadian times (CT00= medium change), OSCs were irradiated (Irr) with a dose of 2 Gy or 10 Gy (n=5/time point in each dose) or handled similarly but not irradiated. 48 hours later, OSCs were collected at the same CTs. Representative photomicrographs of Ki67 immunoreaction in NTL (**A**) and HCC (**B**). Quantification of Ki67 immunoreactive (+) cells in NTL (**C**) and HCC (**D**). Plotted are the mean numbers ± SEM of immunoreactive (+) cells. *: *p*< *0.05* differences between the non-irradiated and irradiated OSCs with a dose of 10 Gy. Scale bars, 100 μm.

In non-irradiated NTL, the number of γ-H2AX+ cells was low and showed a peak at CT02 (Fig. S5C). In HCC, the number of γ-H2AX+ cells was significantly higher in the HCC as compared with NTL (Fig. S5B, D, *p< 0.0001*), and higher at CT14 (*p< 0.05*) and CT20 (*p< 0.01*) as compared with CT08 (Fig. S5D).

In NTL, irradiation with either 2 or 10 Gy resulted in a time-dependent increase in the number of γ-H2AX+ cells (Fig. S5A, C). The strongest effects were observed at CT02 (39.3% and 60.2%, respectively) and CT14 (24% and 27.7%, respectively) (*p< 0.0001*). A smaller effect of irradiation with 2 and 10 Gy was observed at CT08 with a significant increase of 18.4% and 14.5%, respectively (*p< 0.01*). Irradiation at CT20 had no effect on the number of γ-H2AX+ cells (*p> 0.05*). In HCC, the number of γ-H2AX+ cells was further increased after irradiation with 2 or 10 Gy at CT02 (24.8% and 58.4%, respectively) and CT08 (19.3% and 20.2 %, respectively) as compared with non-irradiated HCC (*p< 0.0001*). At CT14, only irradiation with 10 Gy resulted in a significant increase in the number of γ-H2AX+ cells as compared with non-irradiated HCC (17%, *p< 0.001*). At CT20, irradiation had no effect on the number of γ-H2AX+ cells (*p> 0.05*, Fig. S5B, D).


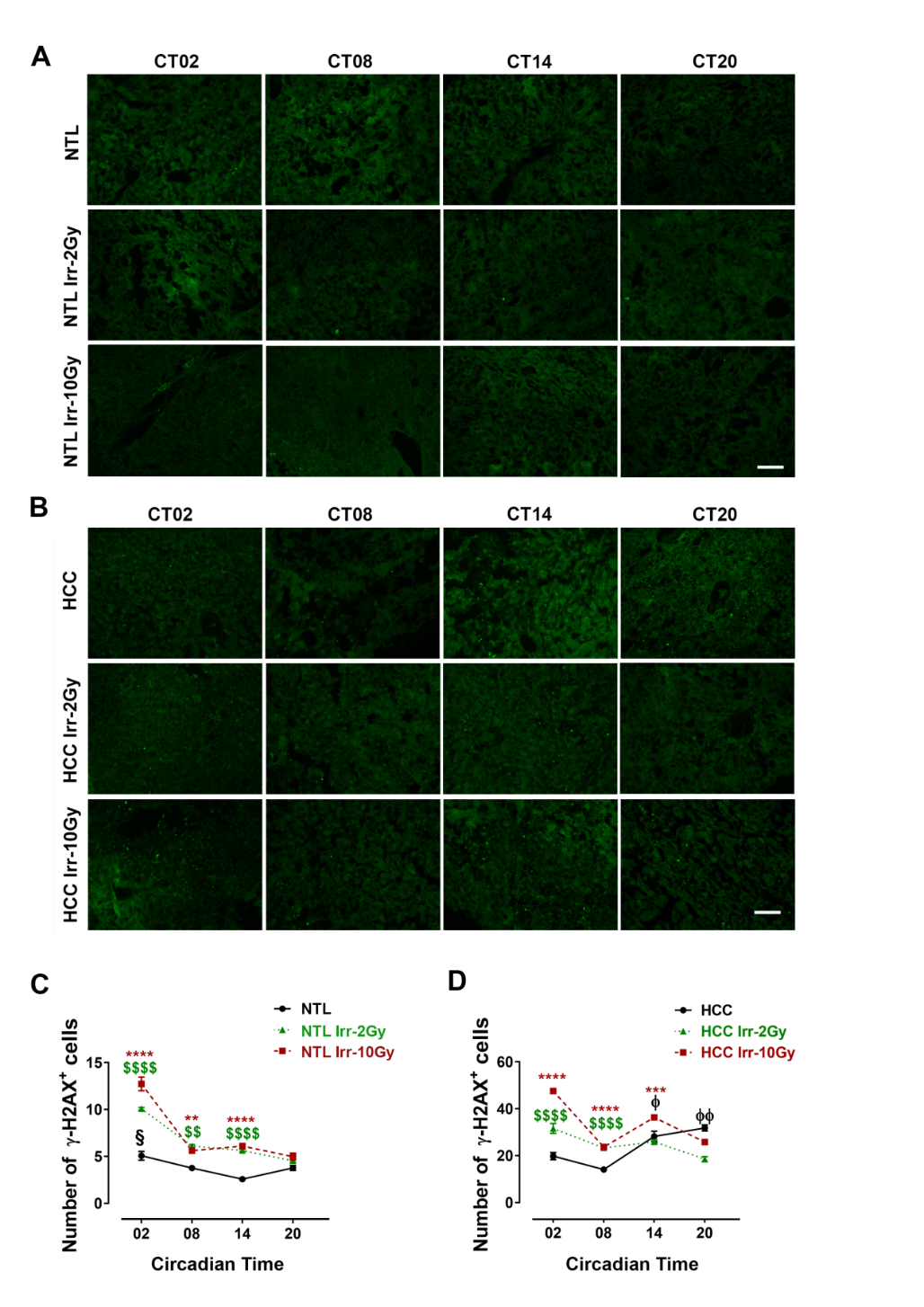


**Figure S5.** γ-H2AX in organotypic slice cultures (OSCs) of hepatocellular carcinoma (HCC) and surrounding non-tumoral liver (NTL) with or without irradiation. At different circadian times (CT00= medium change), OSCs were irradiated (Irr) with a dose of 2 Gy or 10 Gy (n=5/time point in each dose) or handled similarly but not irradiated. 48 hours later, OSCs were collected at the same CTs. Representative photomicrographs of γ-H2AX immunoreaction in NTL (**A**) and HCC (**B**). Quantification of γ-H2AX immunoreactive (+) cells NTL (**C**) and HCC (**D**). Plotted are the mean numbers ± SEM of immunoreactive (+) cells. §: *p*< *0.05* differences between this CT and CT14. φ: *p*< *0.05*; φφ: *p*< *0.01* differences between this CT and CT08.**: *p*< *0.01*; ***: *p*< *0.001*; ****: *p*< *0.0001* differences between the non-irradiated and irradiated OSCs with a dose of 10 Gy. $$: *p*< *0.01*; $$$$: *p*< *0.0001* differences between the non-irradiated and irradiated OSCs with a dose of 2 Gy. Scale bars, 100 μm.


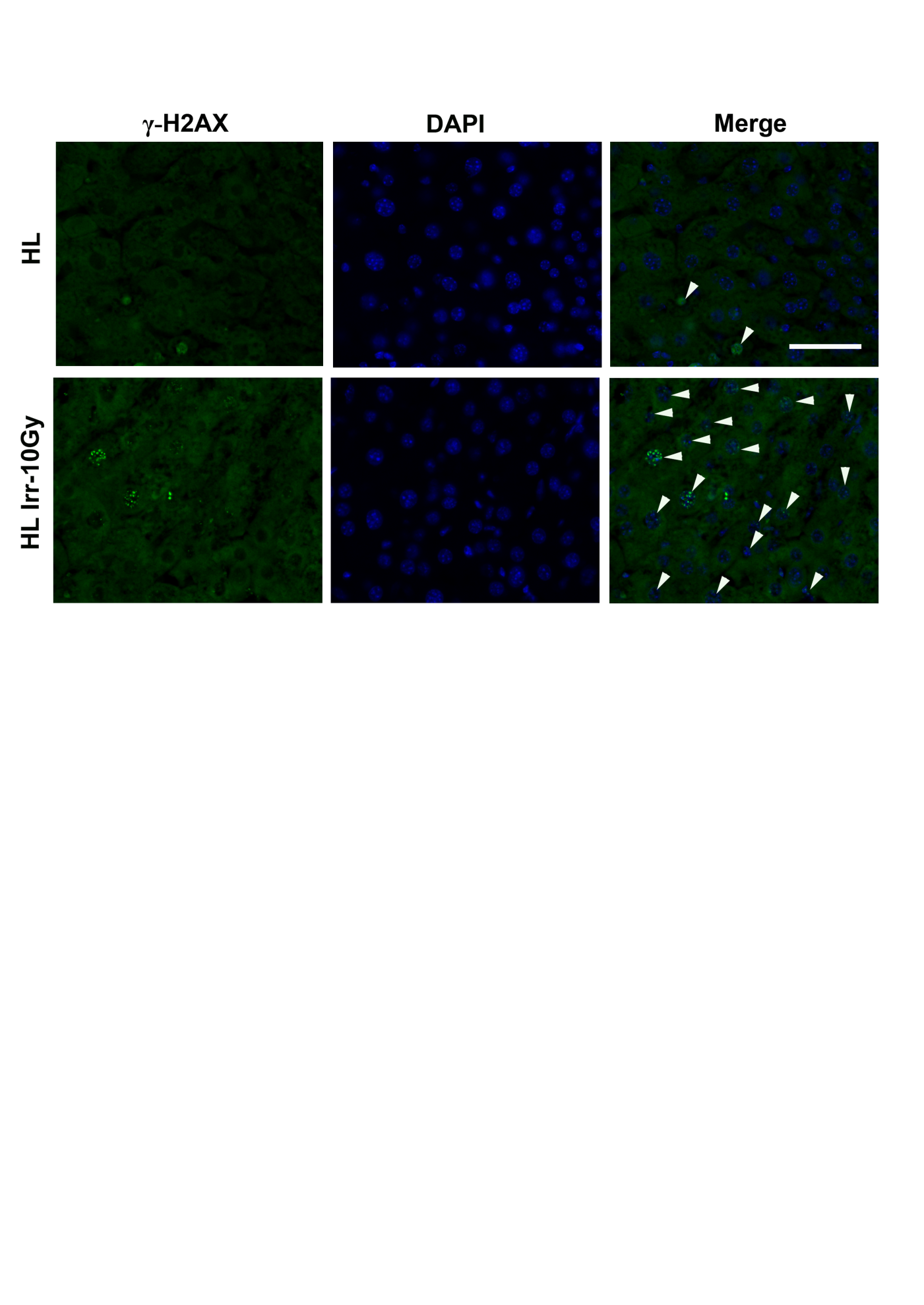


**Figure S6.** Representative high magnification photomicrographs of γ-H2AX immunoreactive (+) (green) in DAPI stained nuclei (blue) in healthy liver (HL) without or with irradiation (Irr-10Gy) at ZT02. γ-H2AX + cells were defined by co-localization of γ-H2AX foci (arrows) and DAPI. Scale bar, 50 μm. .


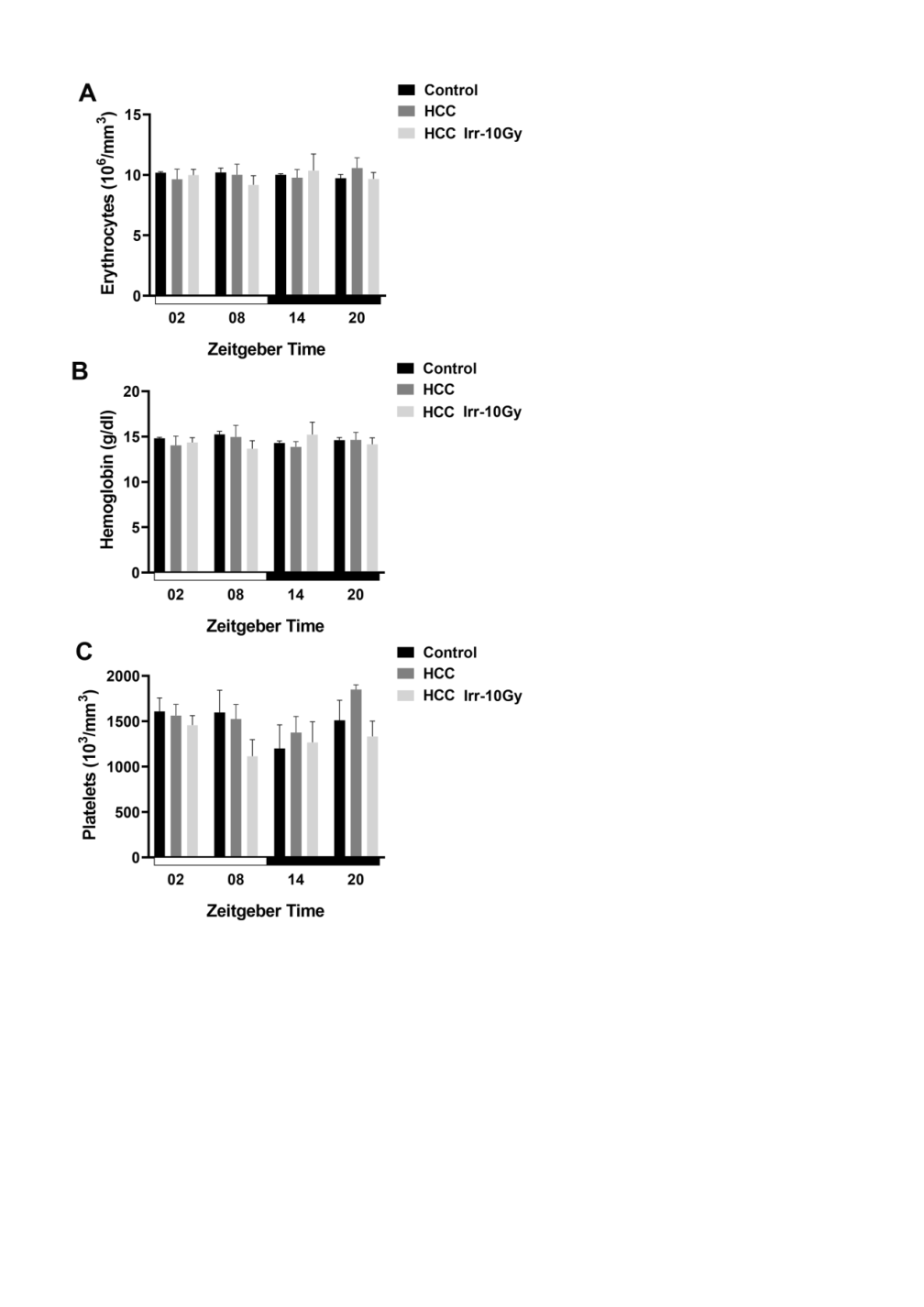


**Figure S7.** Blood cell analysis in control and hepatocellular carcinoma (HCC) bearing mice without and with irradiation. At different *Zeitgeber* times (ZT00= the onset of the light phase), mice were irradiated (Irr) with a dose of 10 Gy (n= 3-6/time point) or handled similarly but not irradiated. 48 hours later, mice were sacrificed and the blood was collected at the same ZTs. Erythrocyte numbers (**A**). Hemoglobin concentration (**B**). Platelet numbers (**C**). Plotted are the mean numbers ± SEM. White and black bars indicate the light and dark phases, respectively.
